# DGOMapping: Real-Time Multi-Agent Mapping Based on 4D Gaussian Splatting

**DOI:** 10.3390/s26123871

**Published:** 2026-06-18

**Authors:** Yonghao Li, Fan Wu, Ping Ye, Qingxuan Jia

**Affiliations:** 1 School of Intelligent Engineering and Automation, Beijing University of Posts and Telecommunications, Beijing 100088, China; yonghaoli@bupt.edu.cn (Y.L.); yeping@bupt.edu.cn (P.Y.); qingxuan@bupt.edu.cn (Q.J.); 2 School of Electronic Engineering, Beijing University of Posts and Telecommunications, Beijing 100088, China

**Keywords:** multi-agent mapping, 4D Gaussian Splatting, dynamic scene reconstruction

## Abstract

Multi-agent perceptual map construction and long-term maintenance constitute an important paradigm for improving adaptability and real-world applicability. With the outstanding capability of 3D Gaussian Splatting in preserving fine-grained texture details, a number of 3DGS-based real-time mapping approaches have recently emerged. However, these methods often struggle to cope with complex dynamics in real-world environments and lack the generalization needed to scale to multi-agent systems. Existing solutions typically rely on direct parameter concatenation or locally confined optimization, which are unable to explicitly model cross-agent observation reliability under temporal asynchrony and dynamic inconsistency, and therefore tend to amplify conflicting updates rather than resolve them. To address these limitations, we propose DGOMapping, an online system for multi-agent dynamic perceptual mapping. DGOMapping leverages an uncertainty-coupled 4DGS scene representation and a collaborative interaction mechanism via Gaussian perception-score exchange, enabling both real-time 4DGS construction and long-term map memory adjustment. Experiments on multiple real-world datasets demonstrate that DGOMapping effectively suppresses dynamic interference and exploits multi-agent collaboration, achieving state-of-the-art performance in both tracking and reconstruction. The proposed system therefore provides a practical sensing-oriented solution for collaborative perception and real-time dynamic environment mapping.

## 1. Introduction

Multi-agent perceptual map construction and long-term maintenance are key technical foundations for enhancing an intelligent system’s environmental adaptability, the integrity of spatial cognition, and the reliability of sustained operation [1,2,3]. In complex real-world environments, a single agent is often constrained by viewpoint coverage, occlusions, and dynamic interference, making it difficult to obtain a stable and consistent global scene representation; in contrast, multi-agent systems can significantly improve spatial coverage and redundant observation capability through collaborative perception, providing important support for building high-precision environment maps that can be sustainably updated [4,5,6]. However, online mapping under multi-agent settings not only faces the challenge of fusing multi-source heterogeneous observations, but must also simultaneously cope with practical challenges such as dynamic objects, temporal asynchrony, and map degradation during long-term operation [7,8,9].

In recent years, 3D Gaussian Splatting (3DGS), as an efficient explicit scene representation, has demonstrated significant advantages in rendering quality and real-time performance, and has gradually been introduced into online perception and mapping tasks [10,11,12]. Compared with traditional sparse or voxel-based representations, 3DGS can represent rich geometric and texture details with a compact set of parameters, providing a new technical route for real-time dense mapping [13,14]. Meanwhile, extending Gaussian representations to dynamic scenes has also attracted increasing attention, such as 4D Gaussian Splatting (4DGS) for real-time dynamic scene modeling and rendering [15,16]. However, existing methods based on 3DGS or 4DGS mostly focus on single-agent or weakly dynamic scenes, and typically rely on simple parameter concatenation or local optimization strategies. When these methods are directly extended to multi-agent dynamic environments, problems such as dynamic interference being incorrectly solidified, local noise spreading across the global map, and the map continuously expanding over time often arise, severely limiting their long-term deployment in real-world systems [17,18,19]. This limitation is not merely an empirical phenomenon, but stems from a deeper methodological mismatch between existing designs and the nature of multi-agent dynamic perception. Direct parameter concatenation implicitly assumes that observations from different agents are mutually compatible and equally trustworthy at the moment of fusion; however, in multi-agent dynamic scenes, observations are inherently asynchronous, incomplete, and reliability-imbalanced. As a consequence, once transient dynamics or noisy local updates are encoded as Gaussian parameters, naive stitching has no internal mechanism to distinguish static structure from short-lived disturbance, and erroneous parameters can be directly absorbed into the global map. Likewise, local optimization strategies are restricted to short temporal windows or single-agent neighborhoods, and therefore lack explicit cross-agent consistency constraints and a unified reliability representation. They may reduce residuals locally, but cannot prevent unreliable updates from propagating to the global map after cross-agent interaction. This contradiction becomes more severe in multi-agent 4DGS because 4D Gaussian representations are high-dimensional and temporally coupled: dynamic modeling depends on temporally coherent parameter evolution, while multi-agent collaboration introduces asynchronous observations and communication delays. Therefore, the challenge is not only how to represent dynamic scenes with 4DGS, but also how to endow the representation with reliability-aware fusion and long-term maintainability under asynchronous multi-agent observations.

**Figure 1:**
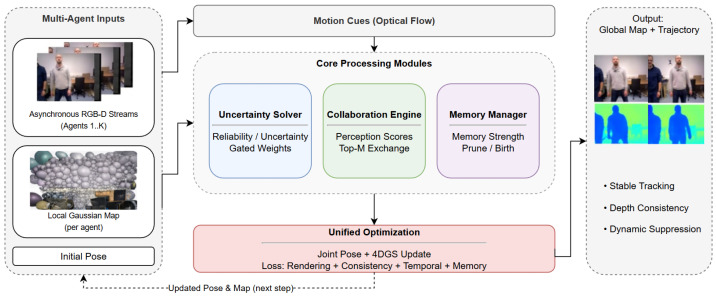
Overview of our multi-agent dynamic-aware 4D Gaussian mapping framework. Asynchronous RGB-D streams are processed by three core modules (uncertainty, collaboration, and memory) and integrated with optical-flow motion cues in a unified optimization. The optimizer jointly refines camera poses and uncertainty-coupled 4DGS parameters under a unified loss, and feeds the updated pose/map back to the next step to form a closed loop. The right panel shows typical outcomes, including stable tracking, consistent depth across frames, and reduced dynamic interference.

To address the above issues, this paper argues that multi-agent 4DGS map construction cannot simply rely on “output-level” stitching or synchronization, but requires systematic mechanism design from three levels: representation, collaboration, and memory [1,7,20]. These three levels correspond exactly to three coupled technical questions in multi-agent dynamic mapping: how to quantify whether a Gaussian observation is reliable, how to let reliable observations dominate cross-agent fusion without parameter conflicts, and how to preserve only repeatedly supported structures during long-term operation. Accordingly, the proposed DGOMapping framework organizes uncertainty coupling, perception-score collaboration, and memory adjustment into a progressive pipeline rather than three isolated modules. Based on this understanding, this paper proposes DGOMapping, a 4DGS mapping framework for multi-agent online dynamic perception and long-term maintenance, as illustrated in Figure 1. The framework integrates three tightly coupled modules—uncertainty coupling, perception-score collaboration, and memory adjustment—into a progressive pipeline, where asynchronous RGB-D observations from multiple agents are jointly optimized with motion cues to update camera poses and 4DGS parameters in a closed loop. The three modules respectively answer the three technical questions raised above, and their designs are detailed in the following three aspects.

To tackle the problems that multi-agent observations exhibit asynchrony and reliability differences, and that direct fusion can easily amplify dynamic noise, this paper proposes an uncertainty-coupled multi-agent 4DGS spatiotemporal scene representation method [5,18,21]. This method explicitly introduces geometric uncertainty variables into the 4DGS representation, and performs online estimation and updates through multi-agent geometric consistency residuals, mathematically characterizing the credibility of each Gaussian primitive under multi-source observations. Furthermore, the uncertainty is directly coupled into rendering and optimization weights, enabling dynamic or conflicting structures to be adaptively down-weighted during multi-agent joint mapping, thereby avoiding error propagation caused by naive parameter concatenation.

To address the issues that directly synchronizing Gaussian parameters among multiple agents leads to high communication overhead, severe conflicting updates, and is unfriendly to asynchronous observations, this paper proposes a collaborative Gaussian perception-score interaction mechanism [4,7]. This mechanism does not enforce consistency at the parameter level; instead, it introduces a perception score for each agent’s observation of Gaussian primitives and uses the scores to drive the generation of cross-agent fusion weights, thereby realizing a collaborative update process in which “high-confidence observations dominate and low-confidence observations are weakened”. In this way, multiple agents can achieve consistent mapping results in dynamic environments in a differentiable and stable manner, while significantly reducing communication and synchronization costs.

To address the problems of map-scale expansion, accumulation of dynamic contamination, and structural degradation during long-term operation of multi-agent systems, this paper proposes a multi-agent 4DGS map memory adjustment mechanism for long-term maintenance [22,23]. This method introduces a memory-strength variable for each Gaussian primitive, and its evolution process is jointly driven by cross-agent support and uncertainty, providing a unified dynamical-systems description of the growth, consolidation, and forgetting of primitives in the map. Through an explicit birth–death mechanism, the system can maintain map compactness and long-term structural stability while continuously performing online mapping, fundamentally distinguishing it from freezing-based or periodic reconstruction strategies. Moreover, this work directly matches the focus of the special issue by addressing how multi-agent sensor observations can be fused into a robust and continuously maintainable perceptual representation for complex dynamic environments.

The contributions of our method can be summarized as follows:

- We construct a unified 4D Gaussian representation framework for multi-agent online mapping, organically integrating temporal evolution, uncertainty evaluation, and explicit Gaussian modeling, so that the scene representation possesses adjustable stability and robustness under asynchronous multi-agent observations.
- We propose a perception-score-based multi-agent collaborative mapping mechanism, using Gaussian primitives as the interaction carrier and achieving consistent cross-agent updates via score-generated fusion weights, thereby avoiding the accumulation of conflicts caused by traditional parameter-level synchronization in dynamic scenes.
- We design a map memory management method for long-term operation, realizing adaptive growth and cleanup of the map structure through modeling and regulation of the memory strength of Gaussian primitives, thus maintaining controllable map scale and structural stability during continuous online updates.

## 2. Related Work

### 2.1. Dense Scene Representation and 3D Gaussian Splatting

Dense scene representation is a core foundation for high-fidelity 3D reconstruction and novel view synthesis [10,24]. Traditional SLAM systems mostly adopt sparse feature points, semi-dense depth maps, or voxel grids as map representations; while such representations are advantageous for robust localization, they are often insufficient to support high-quality rendering and complex scene understanding [22,25,26]. To this end, researchers have begun to introduce continuous scene representation methods, among which implicit representations exemplified by Neural Radiance Fields (NeRF) have achieved remarkable progress in novel view synthesis and geometric modeling [24,27,28]. By learning a continuous mapping from spatial positions and viewing directions to color and density, NeRF enables high-quality dense reconstruction and has been gradually applied to 3D modeling tasks in indoor and controlled scenes [29,30].

However, NeRF-like methods typically rely on volume rendering and backpropagation-based neural optimization, leading to considerable computational and memory overhead, which imposes limitations on real-time performance and long-term online operation [24,29,31]. To overcome these issues, 3D Gaussian Splatting (3DGS) has been proposed as an explicit dense representation [10]. This method models scenes with anisotropic Gaussian primitives and achieves efficient rendering through differentiable splatting, significantly improving computational efficiency while maintaining rendering quality [10]. The advent of 3DGS demonstrates the strong potential of explicit Gaussian representations for real-time novel view synthesis and dense reconstruction [10]. Subsequently, extensive research has been conducted on Gaussian initialization, parameter optimization, rendering stability, and memory compression, gradually making 3DGS feasible as an online map representation [11,12,32].

Furthermore, some works have started to incorporate the temporal dimension into Gaussian representations by modeling dynamic scenes with time-dependent parameters, deformation fields, or temporal regularization, forming 4D Gaussian Splatting or deformable Gaussian models. These methods have made progress in dynamic object modeling and temporal consistency [33,34]; however, their research focus is mainly on dynamic reconstruction under single-view or single-subject settings, with relatively limited attention to unified modeling and stability under multi-source heterogeneous observations [15].

### 2.2. 3DGS-Based SLAM and Mapping in Dynamic Scenes

As the advantages of 3DGS representations in rendering efficiency and expressive capability have become increasingly evident, researchers have begun to incorporate them into simultaneous localization and mapping (SLAM) frameworks to enable high-fidelity dense mapping [11,32,35]. Such methods typically combine conventional pose estimation modules with explicit 3DGS map representations, and perform online or quasi-online scene reconstruction by alternately optimizing camera poses and Gaussian parameters [11,32]. Some works initialize Gaussian primitives using RGB-D sensors or multi-view geometry results and achieve favorable mapping performance in static or weakly dynamic environments [11,32]; other studies reduce the computational complexity of global optimization through submaps, keyframes, or local optimization strategies, thereby improving real-time performance [12,35].

To address the ubiquitous dynamic interference in real-world environments, some studies have started to introduce dynamic handling mechanisms into GS-SLAM frameworks [17,18,19]. For example, by incorporating temporal consistency constraints, dynamic region detection, or Gaussian deformation models, these methods mitigate the impact of dynamic objects on pose estimation and map optimization [18,19,21]. While such approaches improve mapping quality in dynamic scenes to a certain extent, their dynamic modeling often relies on local heuristic strategies and lacks a unified characterization of observation reliability [1]. As a result, they can suppress dynamic interference within a local optimization window, but they do not explicitly explain how reliability should be propagated across agents or across time once the scene representation itself becomes the carrier of collaborative updates.

Overall, existing 3DGS-based SLAM methods mostly focus on single-agent systems and assume relatively consistent observation quality [11,32,35]. When directly extending these methods to multi-agent systems, differences among agents in time, viewpoint, and noise levels can lead to conflicts in Gaussian parameters, amplification of dynamic noise, and degradation of map consistency. More fundamentally, parameter-level fusion treats Gaussian parameters as ready-to-merge outputs, but does not model the asynchronous generation process and reliability difference behind these parameters; local optimization reduces local residuals, but lacks cross-agent consistency constraints and therefore cannot prevent local noise from being broadcast into the shared map. This issue indicates that relying solely on parameter-level fusion or local dynamic suppression strategies is insufficient to support stable mapping in complex multi-agent dynamic environments [4,7,8].

### 2.3. Multi-Agent Collaborative Mapping and Long-Term Map Maintenance

Multi-agent SLAM has long been considered an important means to improve system coverage, robustness, and efficiency. Traditional multi-agent SLAM methods typically achieve collaborative mapping by sharing feature points, pose graphs, or local submaps, and maintain global consistency via centralized or distributed graph optimization [4,7]. These methods are mature in terms of geometric localization accuracy and system stability; however, their map representations are mostly sparse or structured, making them difficult to directly extend to high-fidelity dense representations [22,26].

In recent years, with the development of neural representations and explicit Gaussian representations, some studies have begun to explore dense or neural mapping under multi-agent settings, for example by sharing implicit network parameters, fusing local submaps, or performing unified global optimization on a server to enable collaborative perception [8,9]. However, such methods often implicitly assume similar observation quality across agents, or rely on direct map stitching and parameter synchronization strategies, which tend to cause accumulated conflicts and map inflation under communication constraints, asynchronous observations, and dynamic interference [4,7,8]. The underlying reason is that these strategies emphasize ‘what to transmit’ or ‘how to align parameters’, but do not explicitly model ‘which transmitted information is reliable enough to survive collaboration’. For multi-agent 4DGS, this omission is especially critical because each primitive carries coupled geometric, appearance, and temporal parameters, and once unreliable updates are synchronized, the error is no longer purely local but becomes part of the shared dynamic representation.

In addition, the problem of map maintenance under long-term operation has also attracted attention [1,2]. Existing works typically adopt map freezing, periodic reconstruction, or heuristic redundancy removal strategies to control map scale and error accumulation [2,23]. However, these methods lack an explicit modeling of stability differences across structures in the map, making it difficult to achieve adaptive maintenance under dynamic environments and multi-agent settings [1,2]. In summary, current multi-agent mapping methods still exhibit clear limitations in collaboration mechanisms, dynamic suppression, and long-term maintenance, and it is necessary to introduce a more unified and interpretable modeling framework [1]. This is also why our method jointly introduces uncertainty coupling, score-based collaboration, and memory adjustment: the first provides a unified reliability representation, the second converts reliability into conflict-aware collaborative updates, and the third determines whether fused structures should persist in the map over time.

Based on the above analysis, the proposed DGOMapping systematically extends the applicability of 4DGS to multi-agent dynamic map construction from three perspectives: uncertainty modeling, collaborative interaction, and map memory management, providing a new solution for long-term collaborative perception in complex environments [1,4,15].

## 3. Method

Before detailing each component, we first clarify the overall logic of the proposed framework. The core challenge of multi-agent dynamic 4DGS mapping lies in the fact that asynchronous observations from different agents are not equally reliable, and therefore the system must simultaneously answer three tightly coupled questions: which observations should be trusted, how trusted observations from different agents should be fused, and how the global map should be maintained over long-term operation. To this end, the proposed method is organized into three cooperative modules. The uncertainty-coupled representation addresses the first question by estimating the reliability of each Gaussian primitive under multi-source observations and providing reliability-aware weights for optimization; in other words, it defines a common ‘reliability language’ for all subsequent modules. Building upon this reliability basis, the perception-score interaction module addresses the second question by converting reliability into cross-agent fusion weights, so that high-confidence observations dominate collaborative updates while conflicting or asynchronous updates are suppressed before entering the shared map. Finally, the memory adjustment module addresses the third question by using uncertainty and cross-agent support jointly to regulate the long-term birth, consolidation, and removal of Gaussian primitives, thereby deciding whether short-term fused information should become stable map memory. Therefore, the three modules are not independent add-ons, but form a progressive pipeline from reliability estimation, to collaborative fusion, and further to long-term map evolution. This sequential design also explains why all three modules are necessary: without uncertainty, collaboration lacks a principled basis; without collaboration, uncertainty remains local and cannot constrain multi-agent fusion; and without memory adjustment, even reliable short-term fusion cannot guarantee compact and stable long-term maps.

### 3.1. Uncertainty-Coupled Multi-Agent 4DGS Spatiotemporal Scene Representation

**Figure 2:**
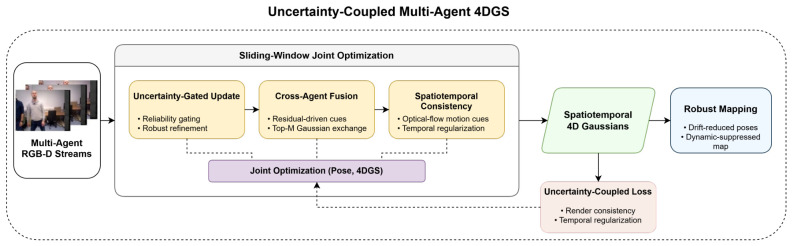
Overview of our uncertainty-coupled multi-agent 4DGS spatiotemporal representation. Asynchronous RGB-D streams are optimized in a sliding window with reliability-gated weighting and cross-agent fusion to suppress dynamic/noisy regions and improve spatiotemporal consistency. This yields more stable pose refinement and a cleaner, long-term map under multi-source observations.

In multi-agent dynamic environments, directly stitching or naively fusing the 3DGS/4DGS maps built by different agents often leads to issues such as dynamic interference being erroneously solidified, local noise spreading globally, and gradual map degradation during long-term operation. The root cause lies in the fact that multi-agent observations are subject to temporal asynchrony, viewpoint discrepancies, and inconsistent noise levels, while conventional explicit representations lack a differentiable modeling of observation reliability and therefore cannot mechanistically distinguish stable structures from transient dynamics. Hence, instead of adopting a direct stitching strategy, we introduce an uncertainty-coupled 4DGS representation from a mathematical perspective to uniformly characterize spatiotemporal consistency and credibility under multi-source observations, and explicitly couple it into rendering and optimization weights, as illustrated in Figure 2.

Let the *k*-th agent capture an image $I_{k,t}$ at time *t*, with pose

(1) $$T_{k,t}=\left[\begin{array}{cc} R_{k,t} & t_{k,t}\\ 0^{⊤} & 1 \end{array}\right]\in SE\left(3\right).$$

We represent the scene by a set of Gaussian primitives evolving over time:

(2) $$M_{t}={\left\{G_{i}\left(t\right)\right\}}_{i=1}^{N_{t}}.$$

Each 4D Gaussian primitive explicitly contains geometry, appearance, and uncertainty variables:

(3) $$G_{i}\left(t\right)=\left(\mu_{i}\left(t\right),\Sigma_{i}\left(t\right),\alpha_{i}\left(t\right),c_{i}\left(t\right),u_{i}\left(t\right)\right),$$

where $\mu_{i}\left(t\right)\in R^{3}$ is the center position, $\Sigma_{i}\left(t\right)\in R^{3\times 3}$ is the anisotropic covariance, which is kept symmetric positive-definite via the scale–rotation factorization $\Sigma_{i}\left(t\right)=R_{i}\left(t\right)S_{i}\left(t\right)S_{i}{\left(t\right)}^{⊤}R_{i}{\left(t\right)}^{⊤}$, $\alpha_{i}\left(t\right)\in \left(0,1\right)$ is the opacity, and $c_{i}\left(t\right)$ denotes appearance parameters (e.g., SH coefficients). The uncertainty vector $u_{i}\left(t\right)\in R^{d_{u}}$ is written as $u_{i}\left(t\right)={\left(u_{i}^{\mathrm{geo}}\left(t\right),\ldots \right)}^{⊤}$, where the scalar $u_{i}^{\mathrm{geo}}\left(t\right)\in R_{\geq 0}$ denotes the geometric uncertainty studied in this paper, and the remaining components (e.g., appearance/semantic uncertainty) are reserved as an extensible interface and not elaborated here. To avoid unconstrained drift induced by the temporal degrees of freedom, and under the assumption that the short-term motion of each Gaussian primitive is locally smooth and can be approximated by a low-dimensional trajectory, we parameterize motion with temporal basis functions:

(4) $$\mu_{i}\left(t\right)=\mu_{i,0}+B\left(t\right)\theta_{i},$$

where $\mu_{i,0}\in R^{3}$ is the initial center position at a reference time $t_{0}$, $B\left(t\right)\in R^{3\times d_{b}}$ is a shared temporal basis matrix (instantiated as linear, B-spline, or low-order polynomial bases, with $d_{b}$ the basis dimension), and $\theta_{i}\in R^{d_{b}}$ are per-primitive learnable coefficients. This parameterization expresses temporal variation while imposing implicit regularization on dynamic complexity. To keep the focus on the core problem of this paper (multi-agent collaboration coupled with uncertainty), the remaining time-dependent quantities $\Sigma_{i}\left(t\right),\alpha_{i}\left(t\right),c_{i}\left(t\right)$ are approximated as piecewise constant within each sliding window, i.e., treated as window-wise time-invariant optimization variables, while only $\mu_{i}\left(t\right)$ evolves explicitly with *t*. Uncertainty is used to characterize the credibility of each primitive under multi-agent observations. Here we assume that reliable static structures produce relatively consistent geometric observations across agents and over time, whereas transient dynamics, occlusions, and mismatches manifest themselves as persistently larger cross-view geometric residuals. Based on this assumption, we define geometric uncertainty $u_{i}^{\mathrm{geo}}\left(t\right)$ and update it via cross-agent residuals. For the observation of agent *k* at time *t*, we project the Gaussian center onto the image plane:

(5) $$p_{i}^{k,t}=\pi \;\left(K_{k}\left(R_{k,t}\mu_{i}\left(t\right)+t_{k,t}\right)\right),$$

where $K_{k}$ is the camera intrinsic matrix and $\pi \left(\cdot \right):R^{3}\;\to \;R^{2}$ denotes the perspective division operator $\pi \left({\left[x,y,z\right]}^{⊤}\right)={\left[x/z,\;y/z\right]}^{⊤}$ (lens distortion is not modeled explicitly, assuming images are pre-undistorted). With the observed depth $D_{k,t}$ (which can come from RGB-D/ToF/multi-view estimation, etc.), we construct the geometric consistency residual:

(6) $$r_{i}^{k,t}={∥D_{k,t}\;\left(p_{i}^{k,t}\right)-\hat{D}\;\left(\mu_{i}\left(t\right);T_{k,t}\right)∥}_{1},$$

where $\hat{D}\left(\mu_{i}\left(t\right);T_{k,t}\right)={\left[R_{k,t}\mu_{i}\left(t\right)+t_{k,t}\right]}_{z}$ is the geometric depth obtained by transforming the Gaussian center into agent *k*’s camera frame and taking the *z*-component. We employ robust statistics and a momentum update to refine the geometric uncertainty:

(7) $$u_{i}^{\mathrm{geo}}\left(t\right)\leftarrow \left(1-\eta \right)\;u_{i}^{\mathrm{geo}}\left(t\right)+\eta \;\rho \;\left(\frac{r_{i}^{k,t}}{\tau_{\mathrm{geo}}}\right),$$

where η∈(0,1) is the momentum coefficient, $\tau_{\mathrm{geo}}>0$ is a scale parameter, and ρ(·) can be instantiated as the Huber or Charbonnier loss (Charbonnier by default in our experiments). Newly spawned primitives are initialized with $u_{i}^{\mathrm{geo}}\left(t_{\mathrm{init}}\right)=u_{0}$ (we set $u_{0}=1$ as a neutral prior, biased toward neither reliable nor unreliable in the absence of evidence). When multiple agents provide simultaneous observations ${\left\{\left(k,t\right)\right\}}_{k\in A_{t}}$ at the same timestamp, the update above is applied sequentially across agent indices, which amounts to an exponential moving average over the current batch of residuals and is insensitive to the update order. To make uncertainty directly affect map updates, we introduce a reliability-gated weight and couple it into rendering weights. We assume a monotonic negative relation between uncertainty and observation reliability, and adopt a Sigmoid mapping to obtain a bounded and differentiable weighting function:

(8) $$w_{i}\left(t\right)=\sigma \;\left(-\gamma_{g}u_{i}^{\mathrm{geo}}\left(t\right)\right)\in \left(0,1\right),$$

where σ(·) denotes the Sigmoid function and $\gamma_{g}>0$ is a temperature coefficient. We finally jointly optimize poses and map parameters within a sliding window W:

(9) $$\min_{\left\{G_{i}\right\},\left\{T_{k,t}\right\}}\sum_{(k,t)\in W}\sum_{p\in \Omega_{k,t}}{∥{\hat{I}}_{k,t}\left(p\right)-I_{k,t}\left(p\right)∥}_{1}\;\cdot \;\omega_{k,t}\left(p\right)+\lambda_{\mathrm{temp}}L_{\mathrm{temp}}+\lambda_{\mathrm{reg}}L_{\mathrm{reg}},$$

where $W=\left\{\left(k,t\right)\;|\;k\in A,\;t\in \left[t_{\mathrm{cur}}-W+1,\;t_{\mathrm{cur}}\right]\right\}$ is a sliding window of length *W* (A being the set of collaborating agents and $t_{\mathrm{cur}}$ the current frame), $\Omega_{k,t}$ is the valid pixel domain of $I_{k,t}$, ${\hat{I}}_{k,t}\left(p\right)$ is rendered by the differentiable 3DGS rasterizer from the current $\left\{G_{i}\right\}$ and pose $T_{k,t}$, and $\lambda_{\mathrm{temp}},\lambda_{\mathrm{reg}}\geq 0$ are balancing coefficients of the regularizers. The pixel weight is aggregated from the Gaussians contributing to that pixel:

(10) $$\omega_{k,t}\left(p\right)=\sum_{i\in V(p)}a_{i}^{k,t}\left(p\right)\;w_{i}\left(t\right),$$

where V(p) is the set of visible Gaussians covering pixel p after projection (sorted from near to far by depth in the camera frame, with index *i* following this ordering), and $a_{i}^{k,t}\left(p\right)$ is the standard 3DGS alpha-compositing weight:

(11) $$a_{i}^{k,t}\left(p\right)=\alpha_{i}\left(t\right)\;G_{i}^{2D}\left(p\right)\;\prod_{j<i}\;\left(1-\alpha_{j}\left(t\right)\;G_{j}^{2D}\left(p\right)\right),$$

in which $G_{i}^{2D}\left(p\right)$ denotes the 2D Gaussian response of the *i*-th primitive at p after projection. Temporal regularization is used to suppress high-frequency jitter:

(12) $$L_{\mathrm{temp}}=\sum_{i}\sum_{t\in W_{t}}\;\left({∥\frac{d\mu_{i}\left(t\right)}{dt}∥}_{2}^{2}\;+\xi {∥\frac{d^{2}\mu_{i}\left(t\right)}{dt^{2}}∥}_{2}^{2}\right)\;,$$

where $W_{t}$ is the set of timestamps appearing in W, ξ≥0 weights the second-order term relative to the first-order one (controlling the penalty on acceleration changes; larger ξ yields smoother trajectories), and continuous derivatives are approximated by finite differences between adjacent timestamps in practice. Parameter regularization suppresses covariance divergence and opacity degeneration:

(13) $$L_{\mathrm{reg}}=\sum_{i}\left(∥\log S_{i}\left(t\right){∥}_{F}^{2}+{∥\alpha_{i}\left(t\right)∥}_{2}^{2}\right),$$

where $S_{i}\left(t\right)=\mathrm{diag}\left(s_{i,1},s_{i,2},s_{i,3}\right)$ is the diagonal scale matrix from the scale–rotation factorization of $\Sigma_{i}\left(t\right)$, and $\log S_{i}\left(t\right)=\mathrm{diag}\left(\log s_{i,1},\log s_{i,2},\log s_{i,3}\right)$ takes the element-wise logarithm of its diagonal entries; this is equivalent to applying an $\ell_{2}$ penalty on the log-scale parameters in the standard 3DGS implementation, preventing Gaussians from elongating or collapsing without bound during optimization. For reproducibility, the hyperparameters introduced in this section take the following default values: momentum η=0.1, geometric scale $\tau_{\mathrm{geo}}=0.05\;m$, temperature $\gamma_{g}=4.0$, temporal-regularizer weight $\lambda_{\mathrm{temp}}=10^{-2}$, parameter-regularizer weight $\lambda_{\mathrm{reg}}=10^{-4}$, second-order smoothness weight ξ=0.1, window length W=8, and initial uncertainty $u_{0}=1$. The above design enables primitives in uncertain, conflicting, or dynamic regions to be adaptively down-weighted during optimization, thereby avoiding error propagation caused by naive multi-agent fusion. More importantly, the estimated uncertainty is not only used for local rendering and pose–map refinement, but also serves as the reliability foundation for subsequent cross-agent collaboration. In this sense, the uncertainty-coupled representation does not solve collaboration by itself; instead, it provides the quantitative basis for deciding which Gaussian observations should play a dominant role during multi-agent interaction, and later also provides one of the key signals for memory update and primitive removal.

### 3.2. Gaussian Perception-Score Interaction Mechanism for Multi-Agent Collaboration

In multi-agent systems, directly synchronizing 4DGS parameters across agents (e.g., averaging or simply stacking Gaussian parameters) not only incurs communication costs that grow linearly with the number of primitives, but also causes conflicting updates in dynamic regions, allowing the global representation to be “hijacked” by low-quality observations. More importantly, multi-agent observations are inherently asynchronous, and enforcing strong parameter-level synchronization amplifies the instability introduced by latency and noise. Therefore, instead of parameter stitching, we design a differentiable *score-driven interaction* mechanism at the decision level: each agent exchanges only perception scores of Gaussian primitives and necessary summaries, and uses these scores to generate fusion weights, thereby realizing the principle that “more credible observations dominate fusion.”

Let the multi-agent communication graph be G=(K,E). For the local observation of agent *k* at time *t*, we define a perception score for each Gaussian. This design is based on the assumption that the usefulness of an observation for collaborative fusion depends jointly on three factors: intrinsic observation quality, actual visibility, and geometric reliability. Under this factorized assumption, the perception score is defined as

(14) $$s_{i}^{k,t}=q_{i}^{k,t}\;v_{i}^{k,t}\;\exp\;\left(-\beta \;u_{i}^{\mathrm{geo}}\left(t\right)\right),$$

where $q_{i}^{k,t}\in \left[0,1\right]$ is an observation-quality term (which can be provided by tracking confidence, exposure/blur assessment, etc.), and $v_{i}^{k,t}$ is a visibility term that can be approximated by the accumulated contribution of this view to the Gaussian:

(15) $$v_{i}^{k,t}=\sum_{p\in \Omega }a_{i}^{k,t}\left(p\right).$$

The uncertainty penalty term naturally assigns lower scores to dynamic or conflicting structures, and thus automatically weakens them during cross-agent fusion.

Agent *k* receives $\left\{s_{i}^{j,t}\right\}$ from its neighbor set N(k) and constructs fusion weights via softmax. This choice is made under the assumption that cross-agent fusion should satisfy two properties simultaneously: observations with higher credibility should contribute more strongly, while all candidate contributions should remain normalized in a differentiable form:

(16) $$\pi_{i}^{k\leftarrow j}\left(t\right)=\frac{\exp\;\left(\kappa s_{i}^{j,t}\right)}{\sum_{\ell \in N(k)\cup \{k\}}\exp\;\left(\kappa s_{i}^{\ell ,t}\right)},$$

where κ>0 controls the “degree of dominance.” Then, we perform a consensus update for Gaussian variables that require alignment (taking the center as an example):

(17) $$\mu_{i}^{k}\left(t\right)\leftarrow \sum_{j\in N(k)\cup \{k\}}\pi_{i}^{k\leftarrow j}\left(t\right)\;\mu_{i}^{j}\left(t\right),$$

and the covariance and appearance parameters are updated in the same manner:

(18) $$\Sigma_{i}^{k}\left(t\right)\leftarrow \sum_{j}\pi_{i}^{k\leftarrow j}\left(t\right)\;\Sigma_{i}^{j}\left(t\right),\;c_{i}^{k}\left(t\right)\leftarrow \sum_{j}\pi_{i}^{k\leftarrow j}\left(t\right)\;c_{i}^{j}\left(t\right).$$

To further suppress cross-agent conflicts in dynamic regions, we introduce a score-weighted consistency constraint so that high-confidence observations enforce stronger consistency:

(19) $$L_{\mathrm{cons}}=\sum_{(k,j)\in E}\sum_{i}\pi_{i}^{k\leftarrow j}\left(t\right){∥\mu_{i}^{k}\left(t\right)-\mu_{i}^{j}\left(t\right)∥}_{2}^{2}.$$

Under communication-limited settings, each agent can transmit only the Gaussian set with Top-*M* scores to reduce bandwidth:

(20) $$S_{k,t}=\mathrm{TopM}\left({\left\{s_{i}^{k,t}\right\}}_{i}\right),$$

and interaction is performed only over $S_{k,t}$, thereby reducing communication and synchronization overhead without sacrificing stability. This mechanism avoids redundancy and conflicts caused by multi-agent parameter stitching, and provides stronger robustness to asynchronous observations. Importantly, the perception-score module is the bridge between uncertainty estimation and long-term maintenance: it transforms local reliability into collaborative decisions and simultaneously produces cross-agent support signals indicating whether a primitive is repeatedly confirmed by different agents. Nevertheless, resolving cross-agent fusion at the current time step is only part of the overall problem, because repeatedly fused observations may still accumulate redundant or unstable structures during long-term operation. Therefore, after establishing reliability-aware collaborative updates, it is further necessary to regulate how these updates are preserved, strengthened, or forgotten over time, which motivates the map memory adjustment mechanism introduced next.

### 3.3. Multi-Agent 4DGS Map Memory Adjustment Mechanism for Long-Term Maintenance

In long-running multi-agent systems, even if unreliable observations are down-weighted locally and cross-agent conflicts are alleviated during short-term collaboration, the global map may still continuously expand over time and gradually accumulate redundancy and contamination caused by transient dynamics, occlusions, and mismatches. This indicates that uncertainty-aware optimization and score-driven collaboration, although necessary, are still insufficient to guarantee long-term map quality on their own. Directly adopting engineered periodic reconstruction or freezing strategies introduces additional computational costs and can easily cause global inconsistency under asynchronous multi-agent conditions. Therefore, as the third component of the framework, we explicitly model a memory strength for each Gaussian primitive, and jointly drive its birth–death behavior and weight adjustment by cross-agent support and uncertainty, thereby transforming short-term reliable fusion into long-term stable map evolution.

We introduce a memory strength $m_{i}\left(t\right)\in \left[0,1\right]$ for each Gaussian primitive, and use cross-agent support as its growth driving force. The underlying assumption is that stable scene structures should be repeatedly supported by multiple agents over time and should simultaneously exhibit low uncertainty, whereas transient or corrupted structures fail to accumulate such consistent support. We first define cross-agent support (a weighted aggregation of scores):

(21) $${\bar{s}}_{i}\left(t\right)=\sum_{k\in K}\omega_{k}\left(t\right)\;s_{i}^{k,t},\;\sum_{k\in K}\omega_{k}\left(t\right)=1,$$

where $\omega_{k}\left(t\right)$ can be given by each agent’s current tracking confidence or a bandwidth allocation policy. The memory strength is updated by a dynamical system with forgetting:

(22) $$m_{i}\left(t+1\right)=\left(1-\lambda_{f}\right)m_{i}\left(t\right)+\lambda_{g}\;\sigma \;\left({\bar{s}}_{i}\left(t\right)-\tau_{s}\right)\;\sigma \;\left(\tau_{u}-u_{i}^{\mathrm{geo}}\left(t\right)\right),$$

where $\lambda_{f}\in \left(0,1\right)$ is the forgetting rate, $\lambda_{g}\in \left(0,1\right)$ is the growth rate, and $\tau_{s}$ and $\tau_{u}$ are the support threshold and the uncertainty threshold, respectively. This update rule ensures that only structures that receive long-term consistent support from multiple agents and have low geometric uncertainty will be gradually consolidated into long-term memory, whereas dynamic or conflicting structures are difficult to accumulate due to low support and/or high uncertainty. Therefore, the memory module is not an isolated post-processing step; it is the final stage of the reliability-to-collaboration-to-maintenance chain, using the uncertainty signal from the first module and the cross-agent support generated by the second module to determine whether a primitive should survive in the map over time.

Based on the above assumption, we use the joint criterion of weak memory and high uncertainty as an operational indicator that a primitive is likely to correspond to unstable or contaminated structure:

(23) $$m_{i}\left(t\right)<\delta_{d}\;\wedge \;u_{i}^{\mathrm{geo}}\left(t\right)>\tau_{d}\;\Rightarrow \;G_{i}\;\mathrm{remove},$$

so that unstable primitives and dynamic contamination can be continuously cleaned during long-term maintenance. Conversely, for persistent regional errors that cannot be explained over time, a birth mechanism is triggered by residuals to supplement new Gaussian primitives. We define the cross-agent averaged rendering residual:

(24) $$\bar{e}\left(p\right)=\frac{1}{\left|K\right|}\sum_{k\in K}{∥{\hat{I}}_{k,t}\left(p\right)-I_{k,t}\left(p\right)∥}_{1},$$

and when $\bar{e}\left(p\right)>\tau_{b}$, candidate Gaussian centers $\mu_{\mathrm{new}}$ (given by depth/multi-view estimation) are generated near the back-projection ray corresponding to p, and initialized as

(25) $$m_{\mathrm{new}}\left(t\right)=m_{0},\;u_{\mathrm{new}}^{\mathrm{geo}}\left(t\right)=u_{0},\;0<m_{0}\ll 1,$$

so that they first participate in optimization as short-term memory, and are then promoted to long-term memory depending on cross-agent support.

Finally, we incorporate long-term maintenance into a unified objective by enforcing that “high memory implies low uncertainty”:

(26) $$\min_{\left\{G_{i}\right\},\left\{T_{k,t}\right\},\left\{m_{i}\right\}}L_{\mathrm{rgb}}+\lambda_{\mathrm{cons}}L_{\mathrm{cons}}+\lambda_{\mathrm{temp}}L_{\mathrm{temp}}+\lambda_{\mathrm{mem}}\sum_{i}m_{i}\left(t\right)\;u_{i}^{\mathrm{geo}}\left(t\right),$$

where $L_{\mathrm{rgb}}$, $L_{\mathrm{cons}}$, and $L_{\mathrm{temp}}$ correspond to the rendering error, cross-agent consistency, and temporal regularization terms, respectively. This memory adjustment mechanism enables the map to undergo an adaptive long-term evolution of “growth–forgetting–cleanup” under multi-agent asynchrony and dynamic interference, fundamentally distinguishing it from direct stitching or freezing-based maintenance strategies.

## 4. Experiments

### 4.1. Datasets

We evaluate our method on five benchmarks, including TUM RGB-D, the BONN RGB-D dynamic dataset, KITTI Odometry, ReplicaMultiagent, and AriaMultiagent. These datasets are selected to cover complementary experimental conditions, including indoor RGB-D mapping, dynamic indoor scenes, outdoor long-trajectory odometry, synchronized multi-agent collaboration, and asynchronous multi-agent collaboration. In all experiments, the input image streams are processed in their original temporal order, and the ground-truth trajectories are used only for quantitative evaluation rather than for pose optimization or map construction.

For indoor single-agent evaluation, we use the TUM RGB-D benchmark. TUM RGB-D is captured in indoor environments using an RGB-D camera and provides ground-truth camera trajectories, which makes it suitable for evaluating both tracking accuracy and dense reconstruction quality. In the trajectory evaluation, we use six freiburg3 sequences: *sit_st*, *sit_xyz*, *sit_half*, *walk_st*, *walk_xyz*, and *walk_half*. These sequences contain different camera motions and dynamic disturbances, including sitting and walking motions, static and translational camera movements, and sequences with more challenging motion patterns. Therefore, they allow us to test whether the proposed uncertainty-coupled 4D Gaussian representation can maintain stable tracking in indoor scenes with different motion intensities. For reconstruction-quality evaluation on TUM RGB-D, we follow the six sequences reported in the quantitative rendering results: *sit_st*, *sit_xyz*, *sit_rpy*, *walk_st*, *walk_xyz*, and *walk_rpy*. These sequences are used to compute PSNR, SSIM, and LPIPS, so that the photometric fidelity, structural similarity, and perceptual quality of the reconstructed views can be evaluated together.

For dynamic indoor evaluation, we use the BONN RGB-D dynamic dataset. Compared with TUM RGB-D, BONN contains stronger dynamic interference caused by moving people and objects, and is therefore more suitable for testing robustness in non-static scenes. We use four BONN sequences in the experiments: *Balloon*, *ps_tracking*, *p_no_box*, and *synchronous*. These four sequences are used for trajectory evaluation with Absolute Trajectory Error, and the same four sequences are also used for reconstruction-quality evaluation with PSNR, SSIM, and LPIPS. The BONN experiments are designed to verify whether DGOMapping can suppress transient dynamic observations during pose–map optimization and avoid incorrectly baking dynamic objects into the reconstructed map.

For outdoor evaluation, we use the KITTI Odometry benchmark. KITTI provides long-range driving sequences with accurate ground-truth poses and differs from indoor RGB-D datasets in terms of scene scale, camera motion, illumination variation, and viewpoint changes. In this paper, we evaluate the method on three KITTI Odometry dynamic sequences: Seq. 00, Seq. 05, and Seq. 06. These sequences are used to test whether the proposed method can generalize from indoor RGB-D scenes to large-scale outdoor environments. The KITTI experiments mainly focus on long-trajectory robustness, large viewpoint changes, and reconstruction stability under outdoor illumination changes.

For multi-agent collaborative mapping, we use ReplicaMultiagent. ReplicaMultiagent provides multi-agent RGB-D streams from indoor scenes with different viewpoints, which allows us to evaluate cross-agent consistency, fused-map quality, and the accuracy–communication trade-off. In the main multi-agent tracking experiments, ReplicaMultiagent is evaluated with two agents, denoted as K=2, on three scenes: *Off-0*, *Apt-0*, and *Apt-1*. We test both synchronized operation with Δt=0 and asynchronous operation with Δt=10 frames, in order to examine the robustness of the proposed score-driven interaction mechanism under temporal offsets between agents. In addition to tracking accuracy, the same three ReplicaMultiagent scenes are used to evaluate merged-map quality, including PSNR, SSIM, LPIPS, and Depth L1. Furthermore, we conduct scalability experiments on ReplicaMultiagent with K=2, K=3, and K=4 agents to analyze how tracking accuracy and communication overhead change as the number of agents increases.

We also include AriaMultiagent in the multi-agent evaluation to further test the proposed method under a different collaborative setting. AriaMultiagent is evaluated with three agents, denoted as K=3, on two scenes: *Room0* and *Room1*. Similar to ReplicaMultiagent, both synchronized operation with Δt=0 and asynchronous operation with Δt=10 frames are considered. This setting is used to evaluate whether DGOMapping remains stable when more agents are involved and when inter-agent observations are temporally misaligned.

Overall, TUM RGB-D and BONN are used to evaluate indoor single-agent tracking and reconstruction under static-to-dynamic conditions, KITTI is used to evaluate outdoor long-trajectory robustness, and ReplicaMultiagent and AriaMultiagent are used to validate collaborative multi-agent mapping. Across these datasets, the experiments cover six TUM RGB-D freiburg3 sequences for tracking, four BONN dynamic sequences, three KITTI Odometry sequences, three ReplicaMultiagent scenes with K=2 agents in the main comparison, two AriaMultiagent scenes with K=3 agents, and additional ReplicaMultiagent scalability settings with K=2, K=3, and K=4 agents. This dataset design enables a comprehensive evaluation of DGOMapping in terms of tracking accuracy, reconstruction quality, dynamic-scene robustness, fused-map consistency, communication efficiency, and scalability to multiple agents.

### 4.2. Implementation Details

Our method is implemented in Python (v3.10, Python Software Foundation, Wilmington, DE, USA) with the PyTorch framework (v2.1.0, Meta AI, Menlo Park, CA, USA; available at https://pytorch.org/, accessed on 18 May 2026). We incorporate CUDA (v11.8, NVIDIA Corporation, Santa Clara, CA, USA) kernels for time-critical Gaussian splatting rasterization and gradient computation. All experiments are conducted on a single NVIDIA GeForce RTX 4090 GPU (NVIDIA Corporation, Santa Clara, CA, USA). Furthermore, we set $\left(\lambda_{\mathrm{cons}},\lambda_{\mathrm{temp}},\lambda_{\mathrm{reg}},\lambda_{\mathrm{mem}}\right)=\left(0.3,0.02,10^{-4},0.2\right)$, η=0.9, $\tau_{\mathrm{geo}}=0.02$, $\gamma_{g}=15$, κ=6, and Top-M=1024, for all datasets.

### 4.3. Baselines and Evaluation Metrics

We compare our method with representative single-agent dense mapping and SLAM baselines. Specifically, we adopt standard 3D-GS [10] and SplaTAM [32] to serve as strong explicit Gaussian-based static mapping baselines. To further evaluate robustness and consistency in dynamic environments, we compare against SC-GS [36], which represents a highly competitive dynamic Gaussian-based SLAM system. In addition, ORB-SLAM3 [22] is adopted as a widely used geometry-driven baseline to provide a reference lower-bound for tracking robustness under motion clutter.

For multi-agent comparisons, we include classical collaborative SLAM frameworks, CCM-SLAM [4] and Swarm-SLAM [37], which represent centralized and decentralized collaboration paradigms, respectively. We further compare with recent multi-agent dense or neural SLAM approaches, including CP-SLAM [38], MAGiC-SLAM [39], MNE-SLAM [8], MCN-SLAM [9], and MANG-SLAM [40]. In addition, we report ORB-SLAM3 [22] in the multi-agent experiments as a classical feature-based reference, where each agent performs independent tracking without explicit dense collaborative map fusion, in order to provide a geometry-driven lower-bound baseline for comparison.

### 4.4. Single-Agent Results

**Figure 3:**
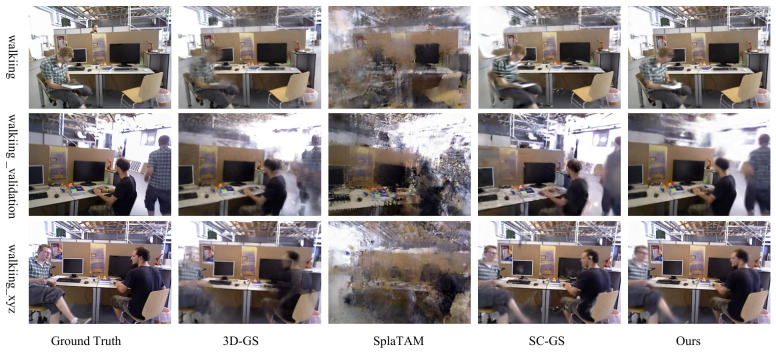
Our results and those of the comparison methods on TUM RGB-D dynamic sequences. We achieve the best performance while ensuring the robustness of tracking.

We first compare mapping and reconstruction quality on the TUM RGB-D benchmark. As illustrated in Figure 3, our reconstructions are visually closer to the ground truth, especially in sequences with frequent camera motion and pronounced dynamic clutter, where both viewpoint changes and occlusions are common. In these challenging cases, the proposed method retains clearer object boundaries and more stable texture details, producing reconstructions that remain consistent across frames rather than fluctuating with transient scene changes. In contrast, existing GS-based SLAM baselines often suffer from either ghosting/blur caused by assimilating transient observations into the map, which leads to duplicated structures and smeared appearances, or local distortions induced by unstable joint optimization under dynamic content, manifesting as warped geometry and inconsistent shading in nearby regions. Benefiting from the proposed uncertainty-coupled 4DGS representation and reliability-aware weighting, our method explicitly down-weights unreliable regions during optimization, thereby suppressing inconsistent dynamic contributions while preserving a sharper and more coherent static background. As a result, the reconstructed renderings exhibit fewer motion-induced artifacts, reduced “dynamic baking” into the static map, and improved visual continuity under rapid motion and heavy clutter.

We then evaluate pose estimation using Absolute Trajectory Error (ATE), with results reported in Table 1 (TUM RGB-D Dynamic), Table 2 (TUM RGB-D Standard), Table 3 (BONN), and Table 4 (KITTI). ATE is computed after trajectory alignment to ground truth and reflects the global deviation accumulated over an entire sequence, making it particularly suitable for assessing robustness under long-term motion and dynamic interference. On TUM RGB-D dynamic sequences (Table 1), our method achieves the best overall accuracy, reducing the average ATE to **2.4 cm** and consistently improving robustness where rapid motion and frequent occlusions typically induce drift.

On standard freiburg3 sequences (Table 2), we observe the same trend: our approach yields the lowest average ATE (**1.61 cm**), outperforming the strongest dynamic baseline SC-GS and remaining stable in challenging indoor scenes where static GS-based methods often degrade due to unconstrained dynamic objects.

On KITTI Odometry, our method further generalizes to large-scale outdoor driving and attains the lowest ATE across the three dynamic sequences, with an average of **0.24 m** (Table 4), indicating improved resilience to long trajectories, varying illumination, and large viewpoint shifts. These gains validate the core design of our optimizer: by down-weighting uncertain regions during pose–map refinement, gradients from dynamic objects are prevented from dominating the update, thereby reducing error accumulation and yielding more stable tracking under strong motion.

**Figure 4:**
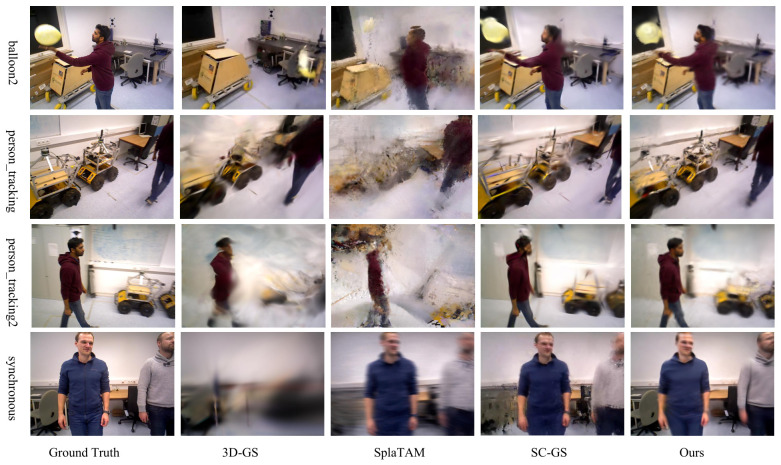
Our results and those of the comparison methods on BONN dynamic sequences. We achieve the best performance while ensuring the robustness of tracking.

As shown in Figure 4, our reconstructed scenes align more closely with the ground truth in terms of geometric accuracy and photometric consistency, particularly in challenging indoor sequences featuring pronounced human motion and object interactions (e.g., fast-moving balloons and active person tracking). In these highly dynamic indoor scenarios, our method successfully preserves precise static background structures (e.g., clear furniture boundaries and wall posters) and consistent texture reproduction across the entire scene, yielding reconstructions that maintain global coherence without transient motion artifacts. In contrast, existing GS-based baselines struggle with two major issues on the Bonn dataset: on one hand, static models like 3D-GS and SplaTAM assimilate dynamic foreground observations into the map, leading to pervasive ghosting, duplicated structures, and severe camera tracking drift caused by dynamic occlusions; on the other hand, while dynamic baselines like SC-GS mitigate drift to some extent, they often induce local distortions and over-smoothed textures in nearby static regions during complex agent-object interactions. Leveraging the proposed uncertainty-coupled 4DGS representation and reliability-aware weighting, our method dynamically adjusts the optimization weights for incoming observations—explicitly down-weighting pixels corrupted by transient human motion or uncooperative clutter while prioritizing structurally confirmed static features. Consequently, our reconstructed renderings exhibit minimal dynamic contamination, highly consistent photometric appearance across varying frames, and enhanced preservation of fine-grained indoor background details, even under persistent environmental disturbances.

Beyond tracking, we assess reconstruction fidelity using PSNR, SSIM, and LPIPS, which jointly measure pixel-level accuracy, structural similarity, and perceptual consistency of rendered views. These metrics provide complementary perspectives on reconstruction quality: PSNR reflects overall radiometric fidelity, SSIM emphasizes local structural preservation, and LPIPS captures perceptual differences that are often aligned with human judgement of visual realism. As shown in Table 5 (TUM RGB-D), our method achieves the best overall rendering quality (PSNR **22.90 dB**, SSIM **0.800** on average), surpassing the strongest baselines and indicating more faithful appearance reconstruction in challenging indoor sequences. On BONN (Table 6), our method again ranks first, obtaining the highest PSNR/SSIM (**24.25 dB**/**0.863**) and the lowest LPIPS (**0.230**), which suggests improved structural coherence and reduced perceptual artifacts under strong dynamic disturbances. On KITTI (Table 7), we observe a similar advantage in outdoor dynamic scenes, where our method achieves the best average reconstruction quality (PSNR **24.42 dB**, SSIM **0.855**, and LPIPS **0.230**), demonstrating that the proposed approach generalizes beyond indoor environments to large-scale driving scenarios. We attribute these consistent improvements to the same mechanism that benefits tracking: suppressing unreliable/dynamic evidence avoids “baking” transient motion into the static map and reduces ghosting in the rendered results, while temporal stabilization regularizes updates across frames, yielding sharper textures, cleaner edges, and fewer motion-induced structural artifacts.

### 4.5. Multi-Agent Results

Table 8 compares multi-agent tracking accuracy on ReplicaMultiagent (K!=!2) and AriaMultiagent (K!=!3) under both synchronized (Δt=0) and asynchronous (Δt=10 frames) operation, thereby explicitly evaluating robustness to inter-agent timing offsets that commonly arise in real deployments. Across all 10 sequences and both synchronization regimes, our method attains the lowest ATE, and the per-dataset averages are improved by 2.6×/2.7× under sync and 2.2×/1.6× under async over the strongest prior method (MCN-SLAM), indicating that the proposed fusion strategy consistently improves global pose estimation across scenes rather than benefiting only a subset.

Notably, the improvement becomes more pronounced under asynchrony, where inconsistent observations and delayed updates tend to amplify drift in collaborative SLAM: on ReplicaMultiagent, the average ATE decreases from 1.01 to 0.23 in the synchronized case and from 1.72 to 0.48 in the asynchronous case (CP-SLAM vs. Ours); on AriaMultiagent, the corresponding averages drop from 2.95 to 0.84 and from 3.90 to 1.64. These results suggest stronger resilience to temporal misalignment and dynamic perturbations, and they are consistent with the proposed reliability-gated cross-agent refinement, which limits the influence of uncertain or rapidly changing regions during joint optimization.

**Figure 5:**
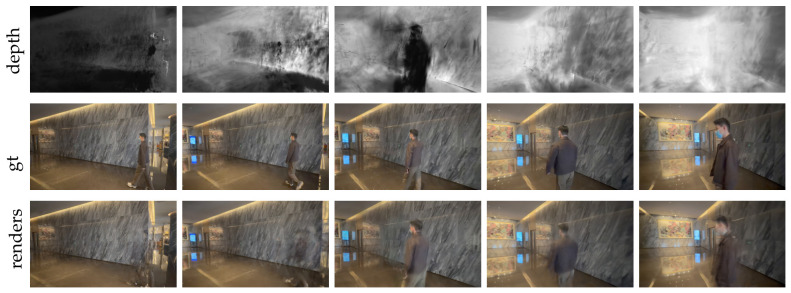
Qualitative results on our self-collected multi-agent dataset. Each column shows a representative frame sampled from the captured trajectory; rows correspond to the predicted depth map (*depth*), the ground-truth depth (*gt*), and the Gaussian-rendered RGB image (*renders*), respectively. The close agreement between predicted depth and ground truth, together with the photorealistic rendered images, demonstrates that our method achieves accurate geometry reconstruction and high-fidelity appearance modelling on real-world multi-agent sequences.

These results suggest stronger resilience to temporal misalignment and dynamic perturbations, and they are consistent with the proposed reliability-gated cross-agent refinement, which limits the influence of uncertain or rapidly changing regions during joint optimization. As a qualitative complement, Figure 5 visualises the depth ground-truth renders on our self-collected dataset, confirming that the reconstructed geometry remains consistent across all agents and sequences.

Beyond tracking, Table 9 evaluates the quality of the fused global map on ReplicaMultiagent, focusing on both rendering fidelity and geometric consistency. Compared with CP-SLAM, our approach improves the average PSNR from 23.4 to 25.1 dB and SSIM from 0.827 to 0.858, while reducing LPIPS from 0.265 to 0.218 and Depth L1 from 4.1 to 2.9 cm, demonstrating that the gain in tracking accuracy translates into a higher-quality reconstructed representation. This suggests that suppressing unreliable evidence during fusion mitigates the “dynamic baking” effect, prevents transient motion patterns from being embedded into the static map, and enhances temporal depth consistency in the merged 4D Gaussian representation across agents and frames.

We further report efficiency and communication overhead in Table 10 to assess practicality under bandwidth constraints. Compared to communication-intensive baselines that require heavy inter-agent map sharing, our method maintains competitive throughput while substantially reducing bandwidth, owing to score-driven Top-*M* exchange rather than full-map synchronization. Finally, Table 11 studies scalability as the number of agents increases. While additional agents introduce more heterogeneous observations and harder cross-agent alignment, our approach scales more gracefully: both ATE and bandwidth grow moderately, whereas the baseline exhibits a sharper increase, particularly under asynchronous operation, indicating poorer robustness to delayed or inconsistent updates.

Overall, compared with both classical collaborative SLAM methods and recent multi-agent neural/Gaussian SLAM baselines, the proposed method consistently achieves the best trade-off among tracking accuracy, fused-map quality, and communication efficiency, making it well suited for online collaborative mapping in dynamic environments.

To more comprehensively validate the proposed framework and to close the loop with the technical challenges analyzed in the Section 3, we further compare representative multi-agent methods from four complementary dimensions: performance, efficiency, robustness, and communication overhead. Specifically, performance reflects whether the method improves the final tracking and mapping quality; efficiency evaluates its suitability for real-time online deployment; robustness measures its sensitivity to asynchronous observations, dynamic disturbances, and communication perturbations; and communication overhead verifies whether the method effectively reduces the cost of cross-agent collaboration. Table 12, Table 13, Table 14 and Table 15 report the corresponding results.

As shown in Table 12, our method achieves the best overall tracking and reconstruction quality, indicating that the proposed uncertainty-aware collaborative optimization improves both geometric consistency and rendering fidelity. Table 13 shows that our method also maintains competitive runtime efficiency, achieving the highest throughput and the lowest mapping latency among the compared methods. In Table 14, our approach exhibits the smallest performance degradation under asynchronous observations, dynamic disturbances, and packet-loss conditions, which is consistent with the intended role of uncertainty coupling and score-driven collaboration in suppressing unreliable updates. Finally, Table 15 confirms that our method incurs the lowest communication overhead, demonstrating that Top-*M* score-driven exchange is more bandwidth-efficient than heavier synchronization strategies. These results jointly show that the proposed framework not only improves final mapping quality, but also offers a better trade-off among accuracy, efficiency, robustness, and communication cost for online multi-agent dynamic mapping.

### 4.6. Ablation Studies

To verify the effectiveness of each module, we construct five ablation variants that progressively remove one design choice at a time to isolate its contribution: **Full** (all modules enabled); **w/o Uncertainty** (removing geometric-uncertainty estimation and reliability gating, i.e., setting all weights to $w_{i}\equiv 1$ so that every observation contributes equally); **w/o Score Interaction** (removing the score-driven interaction mechanism and replacing it with uniform-averaging synchronization across agents); **w/o Memory** (removing memory-strength updates together with the birth/death maintenance strategy); and **Naive Stitching** (direct parameter-level stitching/averaging as a strong yet simplistic baseline). Overall, the results consistently show that each component contributes to robust dynamic mapping: uncertainty gating effectively suppresses dynamic contamination and improves both tracking and rendering consistency; the score-driven interaction alleviates cross-agent conflicts, especially when observations are asynchronous or partially inconsistent; and the memory-adjustment mechanism constrains map growth by preventing redundant accumulation, thereby supporting long-term stability.

Across the BONN *Balloon* and *synchronous* sequences, we conduct ablations to evaluate the contributions of uncertainty coupling, score-driven interaction, and long-term memory adjustment from complementary perspectives (tracking accuracy, efficiency, and reconstruction fidelity). As reported in Table 16, the full model achieves the lowest ATE on both sequences, demonstrating the best overall trajectory accuracy under dynamic disturbances. When uncertainty gating is removed ($w_{i}\equiv 1$), trajectory accuracy consistently degrades on both sequences, indicating that reliability-aware down-weighting is important to suppress dynamic contamination during pose–map refinement and to reduce drift caused by transient motion. Disabling the score-driven interaction and switching to uniform synchronization leads to the largest accuracy drop among the three module removals in Table 16, suggesting that score-based weighting plays a central role in resolving cross-agent inconsistencies and mitigating conflicting updates when observations are not perfectly aligned.

From the efficiency perspective in Table 17, the full model maintains both a compact map and a low communication cost, reflecting the benefits of controlled update strength and selective information exchange. In contrast, removing the memory mechanism noticeably increases the final map size, which is consistent with accumulated redundancy over long-term operation when outdated or low-value primitives cannot be properly decayed or removed. This trend is further mirrored in reconstruction quality: Table 18 shows that the full model yields the best fidelity on both sequences (higher PSNR/SSIM and lower LPIPS), whereas removing uncertainty gating or score interaction leads to a clear drop in visual quality, indicating increased ghosting or structural inconsistency in the reconstructed map. Finally, the naive parameter-level stitching baseline performs worst across ATE (Table 16), reconstruction metrics (Table 18), and communication efficiency (Table 17), highlighting the limitations of direct parameter synchronization and motivating the proposed reliability- and score-aware fusion design.

To validate the contribution of each component, Table 19 reports an ablation over (i) uncertainty-gated weighting, (ii) Top-*M* Gaussian exchange with perception scores, and (iii) motion cues (optical flow), covering both tracking and mapping aspects in the multi-agent setting. Disabling uncertainty coupling degrades both tracking and reconstruction (ATE: 5.1 vs. 3.8; PSNR: 23.6 vs. 25.1), which is consistent with the role of reliability-gated weights in suppressing unreliable or dynamic evidence during joint optimization and preventing such regions from dominating gradient updates. Similarly, removing inter-agent Top-*M* exchange and relying on local-only updates further deteriorates performance (ATE: 5.7; PSNR: 23.2), indicating that selective cross-agent sharing is essential for improving observability, compensating for view-dependent occlusions, and maintaining global consistency across agents.

We further analyze alternative communication designs to clarify the accuracy–bandwidth trade-off. Exchanging Top-*M* Gaussians without scores already improves over local-only fusion (ATE: 4.6; PSNR: 24.1), but it remains inferior to the score-aware variant, suggesting that the perception scores provide an effective mechanism to prioritize informative and reliable Gaussians under limited bandwidth, especially when observations are heterogeneous or partially inconsistent. As a reference upper bound, full Gaussian exchange yields the best accuracy (ATE: 3.5; PSNR: 25.3) but incurs substantially higher communication cost (9.6 MB/s), making it less practical for real-time deployment. In contrast, Top-*M* + scores achieves competitive accuracy with an order-of-magnitude lower bandwidth (1.2 MB/s), thereby offering a more favorable accuracy–communication trade-off. Finally, removing optical-flow cues also reduces both tracking accuracy and rendering fidelity (ATE: 4.4; PSNR: 24.4), confirming that explicit motion information helps enforce spatiotemporal coherence and stabilizes updates under dynamic motion.

Overall, the ablation results support that uncertainty-aware weighting, score-driven Top-*M* interaction, and motion cues are complementary: each contributes to robustness and consistency from a different perspective, and together they enable reliable multi-agent tracking and high-fidelity mapping under practical communication budgets.

## 5. Conclusions

This paper presented DGOMapping, an online multi-agent dynamic perceptual mapping system built upon a 4D Gaussian Splatting representation. To improve robustness under real-world dynamics and asynchronous multi-agent observations, we introduced an uncertainty-coupled 4DGS formulation that explicitly models observation credibility and couples it into rendering and optimization. On top of this representation, we proposed a differentiable Gaussian perception-score interaction mechanism that exchanges lightweight scores rather than full Gaussian parameters, thereby mitigating cross-agent conflicts and reducing communication overhead. Furthermore, we developed a long-term map memory adjustment strategy with a principled birth–death process to control map growth and continuously clean transient contamination, enabling stable long-horizon operation.

Extensive experiments on multiple real-world benchmarks validate that DGOMapping consistently improves both trajectory accuracy and reconstruction quality in dynamic scenes, while maintaining a compact map with low communication cost. Ablation studies further confirm that uncertainty gating, score-driven fusion, and memory adjustment are complementary and jointly contribute to robust multi-agent collaboration. In future work, we plan to extend the framework to more heterogeneous sensor setups and larger-scale agent teams, and to explore tighter integration with loop closure and global consistency constraints for long-term deployment.

## Figures and Tables

**Table 1:** Trajectory errors in ATE (cm) on the TUM RGB-D dynamic sequences (lower is better).

Method	Walking	Walking_Validation	Walking_xyz	Avg.
3D-GS [10]	75.4	68.2	92.5	78.7
SplaTAM [32]	42.3	35.1	58.2	45.2
SC-GS [36]	7.2	5.5	12.1	8.3
Ours	**2.1**	**1.8**	**3.4**	**2.4**

*Note*: Bold values indicate the best performance for each metric.

**Table 2:** Trajectory errors in ATE (cm) on the TUM RGB-D freiburg3 sequences (lower is better).

Method	Sit_st	Sit_xyz	Sit_Half	Walk_st	Walk_xyz	Walk_Half	Avg.
3D-GS [10]	0.75	1.85	25.40	95.20	162.30	155.40	73.48
SplaTAM [32]	0.52	1.60	10.50	80.40	142.50	139.60	62.52
SC-GS [36]	0.50	1.55	4.80	1.85	6.50	6.80	3.67
Ours	**0.49**	**1.40**	**2.40**	**0.67**	**2.20**	**2.50**	**1.61**

*Note*: Bold values indicate the best performance for each metric. Red values indicate revised results or values highlighted for editorial checking in the proof stage.

**Table 3:** Trajectory errors in ATE (cm) on the BONN dynamic sequences (lower is better).

Method	Balloon2	Person_Tracking	Person_Tracking2	Synchronous	Avg.
3D-GS [10]	45.2	82.4	88.7	105.3	80.4
SplaTAM [32]	31.9	76.8	92.9	56.8	64.6
SC-GS [36]	6.8	15.2	8.5	11.1	10.4
Ours	**2.3**	**9.1**	**1.6**	**2.5**	**3.9**

*Note*: Bold values indicate the best performance for each metric.

**Table 4:** Trajectory errors in ATE (m) on the KITTI Odometry dynamic sequences (lower is better).

Method	Seq. 00	Seq. 05	Seq. 06	Avg.
3D-GS [10]	1.45	1.60	1.75	1.60
SplaTAM [32]	0.98	1.05	1.20	1.08
SC-GS [36]	0.42	0.45	0.38	0.41
Ours	**0.24**	**0.27**	**0.22**	**0.24**

*Note*: Bold values indicate the best performance for each metric. Red values indicate revised results or values highlighted for editorial checking in the proof stage.

**Table 5:** Quantitative results on the TUM RGB-D freiburg3 sequences.

Method	Metric	Sit_st	Sit_xyz	Sit_rpy	Walk_st	Walk_xyz	Walk_rpy	Avg.
	PSNR [dB] ↑	18.50	18.20	15.80	14.80	13.50	14.20	15.83
3D-GS [10]	SSIM ↑	0.780	0.730	0.580	0.610	0.490	0.470	0.610
	LPIPS ↓	0.350	0.380	0.580	0.550	0.680	0.690	0.538
	PSNR [dB] ↑	23.55	22.65	20.42	17.25	17.45	16.95	19.71
SplaTAM [32]	SSIM ↑	**0.885**	**0.852**	**0.815**	0.645	0.612	0.595	0.734
	LPIPS ↓	**0.145**	**0.195**	**0.235**	0.315	0.365	0.385	0.273
	PSNR [dB] ↑	26.45	20.95	19.45	21.45	**20.35**	16.85	20.92
SC-GS [36]	SSIM ↑	0.855	0.655	0.565	0.725	0.625	0.515	0.657
	LPIPS ↓	0.215	0.415	0.545	0.325	0.515	0.585	0.433
	PSNR [dB] ↑	**28.15**	**24.85**	**21.15**	**23.45**	20.15	**19.65**	**22.90**
Ours	SSIM ↑	0.865	0.835	0.765	**0.845**	**0.755**	**0.735**	**0.800**
	LPIPS ↓	0.155	0.215	0.295	**0.235**	**0.315**	**0.365**	**0.263**

*Note*: Bold values indicate the best performance for each metric. Red values indicate revised results or values highlighted for editorial checking in the proof stage. ↑ indicates higher is better, and ↓ indicates lower is better.

**Table 6:** Quantitative results on the BONN dynamic sequences. b2 = balloon2, pt1 = person_tracking, pt2 = person_tracking2, sync = synchronous.

Method	Metric	b2	pt1	pt2	sync	Avg.
	PSNR [dB] ↑	20.10	18.50	19.80	18.20	19.15
3D-GS [10]	SSIM ↑	0.740	0.700	0.710	0.720	0.718
	LPIPS ↓	0.490	0.530	0.550	0.450	0.505
	PSNR [dB] ↑	20.45	19.15	21.45	18.65	19.93
SplaTAM [32]	SSIM ↑	0.755	0.655	0.805	0.755	0.743
	LPIPS ↓	0.235	0.305	0.215	0.245	0.250
	PSNR [dB] ↑	21.65	20.85	21.25	**24.45**	22.05
SC-GS [36]	SSIM ↑	0.715	0.725	0.665	0.765	0.718
	LPIPS ↓	0.465	0.415	0.535	0.445	0.465
	PSNR [dB] ↑	**26.65**	**22.55**	**23.85**	23.95	**24.25**
Ours	SSIM ↑	**0.895**	**0.855**	**0.865**	**0.835**	**0.863**
	LPIPS ↓	**0.215**	**0.265**	**0.205**	**0.235**	**0.230**

*Note*: Bold values indicate the best performance for each metric. ↑ indicates higher is better, and ↓ indicates lower is better.

**Table 7:** Quantitative results on the KITTI Odometry dynamic sequences.

Method	Metric	Seq. 00	Seq. 05	Seq. 06	Avg.
	PSNR [dB] ↑	20.10	19.50	18.80	19.46
3D-GS [10]	SSIM ↑	0.745	0.720	0.690	0.718
	LPIPS ↓	0.370	0.400	0.440	0.403
	PSNR [dB] ↑	22.95	22.10	21.50	22.18
SplaTAM [32]	SSIM ↑	0.815	0.795	0.775	0.795
	LPIPS ↓	0.265	0.295	0.320	0.293
	PSNR [dB] ↑	23.80	23.10	22.50	23.13
SC-GS [36]	SSIM ↑	0.840	0.820	0.800	0.820
	LPIPS ↓	0.240	0.265	0.280	0.261
	PSNR [dB] ↑	**25.10**	**24.35**	**23.80**	**24.42**
Ours	SSIM ↑	**0.875**	**0.855**	**0.835**	**0.855**
	LPIPS ↓	**0.205**	**0.230**	**0.255**	**0.230**

*Note*: Bold values indicate the best performance for each metric. Red values indicate revised results or values highlighted for editorial checking in the proof stage. ↑ indicates higher is better, and ↓ indicates lower is better.

**Table 8:** Multi-agent tracking accuracy.

Method	ReplicaMultiagent (K=2)				AriaMultiagent (K=3)		
	Off-0	Apt-0	Apt-1	Avg.	Room0	Room1	Avg.
*Sync (Δt=0)*							
Swarm-SLAM [37]	1.40	1.80	5.60	2.93	6.45	4.78	5.62
CCM-SLAM [4]	1.10	1.55	4.90	2.52	5.30	4.10	4.70
CP-SLAM [38]	0.65	0.95	1.42	1.01	3.03	2.87	2.95
MAGiC-SLAM [39]	0.46	0.62	0.96	0.68	2.42	2.08	2.25
MNE-SLAM [8]	0.54	0.71	1.10	0.78	2.66	2.31	2.49
MCN-SLAM [9]	0.39	0.55	0.82	0.59	2.18	1.92	2.05
MANG-SLAM [40]	0.58	0.79	1.18	0.85	2.80	2.46	2.63
MonoGS [35]	0.85	1.10	1.95	1.30	3.60	3.05	3.33
ORB-SLAM3 [22]	1.75	2.10	6.20	3.35	7.20	5.40	6.30
**Ours**	**0.28**	**0.15**	**0.25**	**0.23**	**1.05**	**0.62**	**0.84**
*Async (Δt=10 frames)*							
Swarm-SLAM [37]	2.10	2.60	7.40	4.03	8.10	6.30	7.20
CCM-SLAM [4]	1.85	2.30	6.70	3.62	6.95	5.55	6.25
CP-SLAM [38]	1.20	1.55	2.40	1.72	4.10	3.70	3.90
MAGiC-SLAM [39]	0.88	1.02	1.56	1.15	3.10	2.74	2.92
MNE-SLAM [8]	0.96	1.15	1.72	1.28	3.34	2.96	3.15
MCN-SLAM [9]	0.80	0.95	1.44	1.06	2.88	2.51	2.70
MANG-SLAM [40]	1.02	1.24	1.86	1.37	3.48	3.12	3.30
MonoGS [35]	1.35	1.70	2.90	1.98	4.70	4.05	4.38
ORB-SLAM3 [22]	2.60	3.05	8.10	4.58	9.40	7.10	8.25
**Ours**	**0.48**	**0.35**	**0.62**	**0.48**	**1.92**	**1.35**	**1.64**

*Note*: Bold values indicate the best performance for each metric.

**Table 9:** Multi-agent merged-map quality on ReplicaMultiagent.

Method	Metric	Off-0	Apt-0	Apt-1	Avg.
CP-SLAM [38]	PSNR (dB) ↑	23.84	23.12	23.27	23.41
	SSIM ↑	0.84	0.82	0.83	0.83
	LPIPS ↓	0.25	0.28	0.27	0.27
	Depth L1 (cm) ↓	3.92	4.28	4.11	4.10
MAGiC-SLAM [39]	PSNR (dB) ↑	24.73	24.05	24.19	24.32
	SSIM ↑	0.85	0.84	0.84	0.85
	LPIPS ↓	0.22	0.25	0.24	0.24
	Depth L1 (cm) ↓	3.31	3.68	3.49	3.49
MNE-SLAM [8]	PSNR (dB) ↑	24.28	23.82	24.03	24.04
	SSIM ↑	0.85	0.83	0.84	0.84
	LPIPS ↓	0.23	0.25	0.25	0.24
	Depth L1 (cm) ↓	3.48	3.91	3.69	3.69
MCN-SLAM [9]	PSNR (dB) ↑	25.02	24.34	24.47	24.61
	SSIM ↑	0.86	0.85	0.85	0.85
	LPIPS ↓	0.22	0.24	0.23	0.23
	Depth L1 (cm) ↓	3.09	3.47	3.31	3.29
MANG-SLAM [40]	PSNR (dB) ↑	24.12	23.53	23.79	23.81
	SSIM ↑	0.84	0.83	0.83	0.84
	LPIPS ↓	0.24	0.26	0.25	0.25
	Depth L1 (cm) ↓	3.58	4.02	3.81	3.80
**Ours**	PSNR (dB) ↑	**25.63**	**24.82**	**25.04**	**25.16**
	SSIM ↑	**0.87**	**0.85**	**0.86**	**0.86**
	LPIPS ↓	**0.20**	**0.22**	**0.22**	**0.22**
	Depth L1 (cm) ↓	**2.71**	**3.08**	**2.89**	**2.89**

*Note*: Bold values indicate the best performance for each metric. ↑ indicates higher is better, and ↓ indicates lower is better.

**Table 10:** Multi-agent efficiency and communication overhead.

Method	ReplicaMultiagent (K=2)		AriaMultiagent (K=3)	
	FPS ↑	Comm. (MB/s) ↓	FPS ↑	Comm. (MB/s) ↓
*Sync (Δt=0)*				
Swarm-SLAM [37]	5.8	4.8	5.1	5.4
CCM-SLAM [4]	8.2	3.2	7.0	3.6
CP-SLAM [38]	7.5	2.0	6.4	3.0
MAGiC-SLAM [39]	8.8	1.6	7.6	2.4
MNE-SLAM [8]	6.9	2.4	5.8	3.3
MCN-SLAM [9]	7.2	2.2	6.1	3.1
MANG-SLAM [40]	7.8	1.8	6.7	2.7
MonoGS [35]	10.8	0.0	9.4	0.0
ORB-SLAM3 [22]	17.6	0.0	15.8	0.0
Ours	9.6	**0.8**	8.4	**1.4**
*Async (Δt=10 frames)*				
Swarm-SLAM [37]	5.1	5.4	4.6	6.1
CCM-SLAM [4]	7.3	3.6	6.2	4.1
CP-SLAM [38]	6.6	2.4	5.6	3.5
MAGiC-SLAM [39]	8.0	1.8	6.9	2.6
MNE-SLAM [8]	6.1	2.8	5.2	3.8
MCN-SLAM [9]	6.5	2.5	5.5	3.5
MANG-SLAM [40]	7.1	2.0	6.1	3.0
MonoGS [35]	9.5	0.0	8.3	0.0
ORB-SLAM3 [22]	16.1	0.0	14.6	0.0
Ours	8.7	**1.0**	7.6	**1.6**

*Note*: Bold values indicate the best performance for each metric. ↑ indicates higher is better, and ↓ indicates lower is better.

**Table 11:** Scalability w.r.t. the number of agents on ReplicaMultiagent.

Variant	Sync (Δt=0)		Async (Δt=10)	
	ATE ↓	Comm. (MB/s) ↓	ATE ↓	Comm. (MB/s) ↓
K=2				
CP-SLAM [38]	1.01	2.0	1.72	2.4
MAGiC-SLAM [39]	0.68	1.6	1.15	1.8
MCN-SLAM [9]	0.59	2.2	1.06	2.5
Ours	**0.23**	**0.8**	**0.48**	**1.0**
K=3				
CP-SLAM [38]	1.28	3.1	2.10	3.8
MAGiC-SLAM [39]	0.92	2.4	1.48	2.8
MCN-SLAM [9]	0.81	3.1	1.34	3.6
Ours	**0.31**	**1.2**	**0.70**	**1.5**
K=4				
CP-SLAM [38]	1.45	4.4	2.48	5.6
MAGiC-SLAM [39]	1.08	3.2	1.76	3.9
MCN-SLAM [9]	0.95	4.1	1.58	4.8
Ours	**0.39**	**1.6**	**0.86**	**2.0**

*Note*: Bold values indicate the best performance for each metric. ↓ indicates lower is better.

**Table 12:** Performance comparison on multi-agent mapping quality.

Method	ATE ↓	PSNR (dB) ↑	SSIM ↑	Depth L1 (cm) ↓
CP-SLAM [38]	1.01	23.4	0.827	4.1
MAGiC-SLAM [39]	0.68	24.3	0.844	3.5
Ours	**0.23**	**25.1**	**0.858**	**2.9**

*Note*: Bold values indicate the best performance for each metric. ↑ indicates higher is better, and ↓ indicates lower is better.

**Table 13:** Efficiency comparison of different multi-agent mapping methods.

Method	FPS ↑	Mapping Latency (ms/frame) ↓	Peak GPU Memory (GB) ↓
CP-SLAM [38]	7.5	133	15.8
MAGiC-SLAM [39]	8.8	114	14.2
Ours	**9.6**	**104**	**13.6**

*Note*: Bold values indicate the best performance for each metric. ↑ indicates higher is better, and ↓ indicates lower is better.

**Table 14:** Robustness comparison under asynchronous, dynamic, and communication-disturbed conditions.

Method	Async ATE Increase ↓	Dynamic PSNR Drop (dB) ↓	PL ATE ↓
CP-SLAM [38]	0.71	1.85	0.64
MAGiC-SLAM [39]	0.47	1.24	0.42
Ours	**0.25**	**0.78**	**0.21**

*Note*: Bold values indicate the best performance for each metric. ↓ indicates lower is better.

**Table 15:** Communication overhead comparison in multi-agent collaboration.

Method	Comm. (MB/s) ↓	Transmitted Primitives/Step ↓	ATE/Comm. ↓
CP-SLAM [38]	2.0	1850	0.505
MAGiC-SLAM [39]	1.6	1420	0.425
Ours	**0.8**	**1024**	**0.288**

*Note*: Bold values indicate the best performance for each metric. ↓ indicates lower is better.

**Table 16:** Ablation study on the BONN sequences (Balloon and synchronous) in terms of ATE.

Variant	Balloon	Synchronous	Avg.
Full	**2.30**	**2.50**	**2.40**
w/o Uncertainty ($w_{i}\equiv 1$)	4.20	4.80	4.50
w/o Score Interaction (uniform sync)	5.50	6.20	5.85
w/o Memory (no birth/death)	2.80	3.10	2.95
Naive Stitching (param-level)	10.00	12.00	11.00

*Note*: Bold values indicate the best performance for each metric.

**Table 17:** Efficiency indicators on BONN. Map size is the final number of Gaussians; communication is measured as transmitted Gaussians per step.

Variant	Map Size (Final #Gaussians) ↓	Comm. Cost ↓
Full	**1.00×**	**1.00×**
w/o Uncertainty ($w_{i}\equiv 1$)	1.05×	1.00×
w/o Score Interaction (uniform sync)	1.10×	1.80×
w/o Memory (no birth/death)	1.45×	1.00×
Naive Stitching (param-level)	1.60×	2.50×

*Note*: Bold values indicate the best performance for each metric. ↓ indicates lower is better.

**Table 18:** Ablation study on BONN (Balloon and synchronous) in terms of reconstruction quality.

Variant	PSNR [dB] ↑		SSIM ↑		LPIPS ↓	
	Balloon	Sync	Balloon	Sync	Balloon	Sync
Full	**26.65**	**23.95**	**0.895**	**0.835**	**0.215**	**0.235**
w/o Uncertainty ($w_{i}\equiv 1$)	25.10	23.10	0.872	0.812	0.245	0.265
w/o Score Interaction (uniform sync)	24.60	22.60	0.858	0.795	0.265	0.290
w/o Memory (no birth/death)	26.10	23.60	0.889	0.829	0.220	0.245
Naive Stitching (param-level)	22.20	19.80	0.780	0.730	0.360	0.410

*Note*: Bold values indicate the best performance for each metric. ↑ indicates higher is better, and ↓ indicates lower is better.

**Table 19:** Ablation on uncertainty coupling and communication.

Variant (K=3)	ATE ↓	PSNR ↑	Comm. (MB/s) ↓
Top-*M* + scores (ours)	**3.8**	**25.1**	**1.2**
w/o uncertainty-gated weights	5.1	23.6	1.2
w/o Top-*M* exchange (local only)	5.7	23.2	0.0
Top-*M* (no scores)	4.6	24.1	0.8
Full Gaussian exchange (upper bound)	3.5	25.3	9.6
w/o optical flow motion cues	4.4	24.4	1.2

*Note*: Bold values indicate the best performance for each metric. ↑ indicates higher is better, and ↓ indicates lower is better.

## Data Availability

The data presented in this study are openly available.
